# The impact of a blended multidisciplinary training for the management of obstetric haemorrhage in Mbeya, Tanzania

**DOI:** 10.3389/fgwh.2023.1270261

**Published:** 2023-12-07

**Authors:** Bernard Mbwele, Amani Twaha, Kasia Maksym, Matthew Caputo, Delfina D. Mkenda, Helen Halpern, Sylvia Berney, Elias A. Kaminyoge, Mpoki S. Kaminyoge, Mandeep Kaler, Soha Sobhy, Sara L. Hillman

**Affiliations:** ^1^ Department of Epidemiology and Bio-Statistics, University of Dar es Salaam—Mbeya College of Health and Allied Sciences, UDSM-MCHAS, Mbeya, Tanzania; ^2^ Programme Development, Vijiji Tanzania, Mbeya, Tanzania; ^3^ Obstetrics Department, Mbeya Zonal Referral Hospital (MZRH), Mbeya, Tanzania; ^4^ Medical School Building, UCL EGA Institute for Women’s Health, London, United Kingdom; ^5^ Department of Statistics, Institute for Global Health, Northwestern University Feinberg School of Medicine, Chicago, IL, United States; ^6^ Programme Development, Tanzania UK Healthcare Diaspora Association, London, United Kingdom; ^7^ Women’s Health Research Unit, Barts and The London School of Medicine and Dentistry, Queen Mary University of London, London, United Kingdom

**Keywords:** obstetric haemorrhage, blended training, multidisciplinary obstetric care, maternal deaths, simulation, Mbeya, Tanzania

## Abstract

**Background:**

The Maternal Mortality Rate (MMR) in Tanzania is 78 times higher than that of the UK. Obstetric haemorrhage accounts for two-thirds of these deaths in Mbeya, Tanzania. A lack of healthcare providers' (HCPs') competencies has been the key attribute. This study measured the impact on HCP's competencies from a blended training programme on obstetric haemorrhage.

**Methods:**

A “before and after” cohort study was undertaken with HCPs in 4 hospitals in the Mbeya region of Tanzania between August 2021 and April 2022. A multidisciplinary cohort of 34 HCPs (doctors, nurses, midwives, anaesthetists and radiologists) were enrolled on a blended face-to-face and virtual training course. The training was delivered by a multidisciplinary team (MDT) from London, UK, assisted by local multidisciplinary trainers from Mbeya, Tanzania and covered anaesthetic, obstetrics, haematology and sonographic use.

**Results:**

There were 33 HCP in the cohort of trainees where 30/33 (90.9%) of HCPs improved their Anaesthesia skills with a mean score improvement of 26% i.e., 0.26 (−0.009 −0.50), 23 HCPs (69.7%) improved obstetric skills 18% i.e., 0.18 (−0.16 to 0.50), 19 (57.6%), (57.6%) improved competences in Haematology 15%.i.e., 0.15 (−0.33 to 0.87), 20 out of 29 HCPs with ultrasound access (68.8%) improved Sonographic skills 13%.i.e., 0.13 (−0.31 to 0.54). All 33 HCPs (100%) presented a combined change with the mean score improvement of difference of 25% i.e., 0.25 (0.05–0.66). The deaths attributed to obstetric haemorrhage, the mortality rate declined from 76/100,000 to 21/100,000 live births. Actual number of deaths due to obstetric haemorrhage declined from 8 before training to 3 after the completion of the training.

**Conclusion:**

This comprehensive blended training on anaesthetic surgical, haematological, and sonographic management of obstetric haemorrhage delivers a significant positive impact on the detection, management and outcomes of obstetric haemorrhage.

## What is already known on this topic?

Previous studies investigating preventable deaths from obstetric haemorrhage have identified that a large proportion can be attributed to provider-related factors such as incomplete or inappropriate management of bleeding before, during or after birth. Training has been shown to improve such outcomes, however different training modalities have had a variable impact.

## What this study adds

The blended training design, which includes a simulation component, has proven successful in knowledge and skills transfer in the recognition and management of obstetric haemorrhage in Tanzania and could easily be implemented in other low and middle-income countries (LMIC) with engagement from local maternity units.

## How this study might affect research, practice or policy

Scaling up and evaluation of multidisciplinary training programmes using blended learning methods is needed in LMIC to reduce maternal deaths from obstetric haemorrhage. Blended training allows for continuing learning and development of HCP's competencies in obstetric haemorrhage management. It offers the opportunity for engagement and empowerment of local healthcare providers when new clinical techniques are introduced.

## Introduction

Obstetric haemorrhage remains the leading cause of direct maternal mortality worldwide, disproportionately affecting pregnant women in low and middle-income countries (1) where 94% of maternal deaths occur (2). Obstetric haemorrhage accounts for a third of all maternal deaths. Each year, about 14 million women experience postpartum haemorrhage (PPH) resulting in about 70,000 maternal deaths globally (3). PPH, formally defined as blood loss exceeding 500 ml following vaginal birth and 1,000 ml following caesarean, accounts for three-quarters of all obstetric haemorrhage deaths (4). The majority of deaths related to PPH occur within 24 h of delivery, suggesting inherent risks at the time of birth (5, 6).

Maternal deaths in Tanzania (7, 8) are 78 times higher compared to that of the UK (9, 10) with obstetric haemorrhage as the cause in 38% to 69% of cases (11–13). In Mbeya, Southwest Tanzania, only half of pregnant women receive the minimum standard of obstetric care and maternal deaths are 776 per 100,000 live births (14).

Evidence presented by the UK maternal mortality surveillance system MMBRACE identified that improvement in care may have made a difference in outcome in 37% of cases of maternal deaths they investigated (10). The use of multiple interventions to control haemorrhage, including oxytocin, misoprostol, tranexamic acid, new surgical techniques and blood transfusion, are all recommended to help achieve haemodynamic stability (15). Blood loss due to failure of the uterus to contract after birth can be minimised if oxytocin is available and given, but it is clear that many women die shortly after childbirth due to this medication not being readily available or not recognised as useful (16).

The most common causes of Obstetric haemorrhage (OH) are known as “the 4 T's”; uterine aTony, Trauma, Tissue (retained) and coagulopathy (Thrombin) (17). Many of these features are better recognised and managed following a robust multidisciplinary (MDT) training approach and identification of pre-existing risk (18) alongside access to effective intrapartum care of OH (19).

Evidence from Northern Tanzania showed that teams, skills training, and realistic simulated scenarios can reduce OH complications (20). The COVID-19 pandemic made virtual teaching the optimum route of delivery. We therefore sought to test the use of a virtual blended training programme with HCPs in Mbeya, Tanzania. We hypothesized that blended training (instruction/physical simulation) delivered virtually by UK and Tanzanian experts, supplemented by simulation training, alongside structured assessment, would improve HCPs' knowledge and skills in managing massive obstetric haemorrhage (MOH) cases in Mbeya, Tanzania.

## Materials and methods

### Design

A “before and after” cohort study to investigate the effectiveness of blended training for the management of MOH was conducted in four hospitals in Mbeya, Tanzania from the baseline in August 2021 to completion in April 2022. Institutional and Tanzanian ethical approval was obtained for all sites before the commencement of the intervention and data collection.

### Setting

The study was conducted in four hospitals in Mbeya—Mbeya Zonal Referral Hospital (MZRH), Mbeya Regional Referral Hospital (Mbeya RRH), Mbalizi Designated District Hospital (Mbalizi DDH) and Igawilo City District Hospital (Igawilo DH). The project was publicised widely at the hospital sites. Hospital management and stakeholders were engaged before recruitment to facilitate uptake and to ensure that there was support for those enrolled to complete the course. HCPs from the 4 hospitals were approached and self-registration took place in each of the sites supported by a local dedicated study team.

### Participants

Multidisciplinary teams from the four hospitals were recruited from all seven available groups of HCPs involved in caring for pregnant women including; medical doctors (graduate and specialist obstetricians), midwives, nurses, anaesthetists and anaesthetic nurses, laboratory technicians and sonographers.

### Intervention

The intervention consisted of 4 modules: (1) Risk assessment (2) Surgical, Haematological and Medical Treatments for Management (3) Anaesthetic considerations (4) The Role of Ultrasound to Improve Diagnosis.

The training was delivered virtually through an online platform with instructors from University College London (UCL) and Queen Mary University London (QMUL). The platform provided students with a mix of live interactive lectures and practical sessions that were delivered at a set time to each of the four sites, so students could view and engage together. The intervention ran over 10 consecutive weeks with 4 areas covered sequentially. The areas covered included: (1) Risk assessment (2) Surgical, Haematological and Medical Treatments for Management (3) Anaesthetic considerations (4) The Role of Ultrasound to Improve Diagnosis.

Each module contained regular teaching/demonstrations at a specific for the HCPs participating and delivered live through the educational platform. Appropriate resources such as guidelines and protocols were uploaded to the platform so students could review them before and after sessions. These resources were recovered from the World Health Organisation, Royal Colleges and GLOWM websites (21–23). In the risk assessment module students were taught about recognising and initial management of deteriorating patients, A–E assessment, situation, background, assessment and recommendation (SBAR) techniques and how to estimate blood loss. Live lectures were complemented by additional practical demonstrations. All sessions were recorded and uploaded to the platform shortly after airing so students could review the content at their leisure. Module 2 lasted 3 weeks and covered haematological considerations, medical management, basic (B-lynch/ tamponade) as well as advanced surgical skills to manage PPH. Again, sessions were complemented with practicals for B Lynch and balloons (condom) training. The anaesthetic module covered the choice of regional vs. general, airway management and fluid resuscitation. The ultrasound module covered basic ultrasound relevant to the risk of bleeding and practical sessions about equipment and views. All four modules also had a tutorial session at the end in which students fed back short answer responses they had been sent at the beginning of the module and had time to ask questions and clarify details. All modules had pre and post-quiz within the content that required completion to access and move on to the next module.

Additional face-to-face practical training sessions (for surgical techniques) were facilitated by local experts from MZRH who also helped to facilitate the simulation training days in conjunction with the UK training team (interacting virtually).

### Simulation training session

All students undertook one-day simulation training with a pass/fail assessment built in. Students were offered a variety of dates so all could attend at least one session. Advanced life-like mannequins (human patient simulators) were used in simulated scenarios to represent realistic clinical obstetric environments. Participants performed in their clinical roles and communicated via the SBAR tool (24) around emergency obstetric haemorrhage cases and their management through newly learned techniques.

Assessment took place by the UK team observing an end-of-day full clinical scenario in which all HCPs participated in in their normal professional capacities.

### Formal assessment and data collection

Information about risk factors, rates of haemorrhage, morbidity and mortality was collected and verified through onsite visits to mortuaries and by review of case notes from the individual participating hospitals.

Changes in HCP knowledge and skills were assessed through an initial baseline assessment, and online written assessments before and after each module, followed by an end-of-training formal written assessment under exam conditions that covered all components of the course. Data was collected using Epi-Collect 5 software. Assessment scores for individual modules were generated from the pre and post-module scores for each component and overall for the course. Both overall mean score changes and improvement of individual HCP scores were reported. In the final overall assessment, a pass mark of above 75% was required to achieve a certificate of competency. Students who did not make this grade were allowed one further attempt in similar conditions with different examination questions.

In addition to the pre and post-module-based competencies, specific practical and management skills were also assessed including special cases of OH risk, blood loss estimation, blood transfusion practices, use of balloon tamponade, and B-lynch compressive sutures.

### Statistical analysis

Analyses were performed using GraphPad Prism Version 9.4.0 (453) and R 4.1.1. The distribution of data was assessed using the D’Agostino and Pearson test, Anderson–Darling test, Shapiro–Wilk test and Kolmogorov-Smirnov tests for normality vs. lognormality. Ordinary one-way ANOVA, paired and unpaired two-tailed Student t-tests were applied to normally distributed data and the Mann–Whitney test and Kruskal–Walis test was applied to not normally distributed data. Šídák's multiple comparisons test was used to assess the total score difference before and after the module.

Paired pre and -post-test data were analysed for each module, and respondents were only included if they completed both assessments. Individual test score improvement was visualized using connected scatterplots and the distributions of pre and post-test data were visualized with boxplots, separated by module. For each module, the percentage of participants that improved was calculated. 95% confidence intervals for mean improvement, which was calculated as post-training score minus pre-training score, were estimated using a percentile bootstrap with 1,000 bootstrap replicates.

Maternal mortality rates were reported as absolute and calculated as the number of maternal deaths per 100,000 live births. The pre-training and post-training mortality rates were compared for the four hospitals individually, as well as cumulatively from data analysed during the month before and the month after the training. Fisher's exact tests were used for proportion comparison due to small death counts.

## Results

The project targeted a total of 25 HCPs, with a male-to-female ratio of 1:1. However, due to high interest from HCPs, a total of 34 HCPs were enrolled with a distribution of 8 females (23.5%) and 26 males (76.5%). The characteristics of the HCPs recruited are described in Table 1.

**Table 1:** total number of health care providers by cadres, gender and facility of training.

Cadre credential	Freq.	Proportion	Gender	Freq.	Proportion
Anaesthetic nurse	3	8.8	Female	8	23.5
Clinical officer (health assistant)	3	8.8	Male	26	76.5
Lab technician	1	2.9	Facility	Freq.	Proportion
Medical doctor (obstetrician)	11	32.4	ICDH	6	17.7
Midwife	9	26.5	MDDH	9	26.5
Nurse	4	11.8	MRRH	8	23.5
Sonographer	3	8.8	MZRH	11	32.4
	**34**	**100**.**0**		**34**	**100**.**0**

HCPs were from a range of multidisciplinary groups including; anaesthetic nurses working in obstetrics, laboratory technicians, clinical officers, nurses, midwives and doctors including those working specifically in obstetrics. The 34 participants represented 30.8% of all available HCPs in the region (*n* = 107). Importantly, 15 out of 34 (43%) were midwives and anaesthetic nurses working in obstetrics (representing 43% of available staff in the region), or 15 out of 34 (26.5%) were medical doctors and obstetricians representing 13 out of 34 38%) of available HCPs in the region. Staff came from all hospitals with proportionally more from the regional centre, MZRH.

Before the final assessment, one HCP (obstetrician) had to drop out due to a hospital site transfer, so outcome data is reported for the remaining 33 HCPs.

### Overall assessment scores

**Figure 1:**
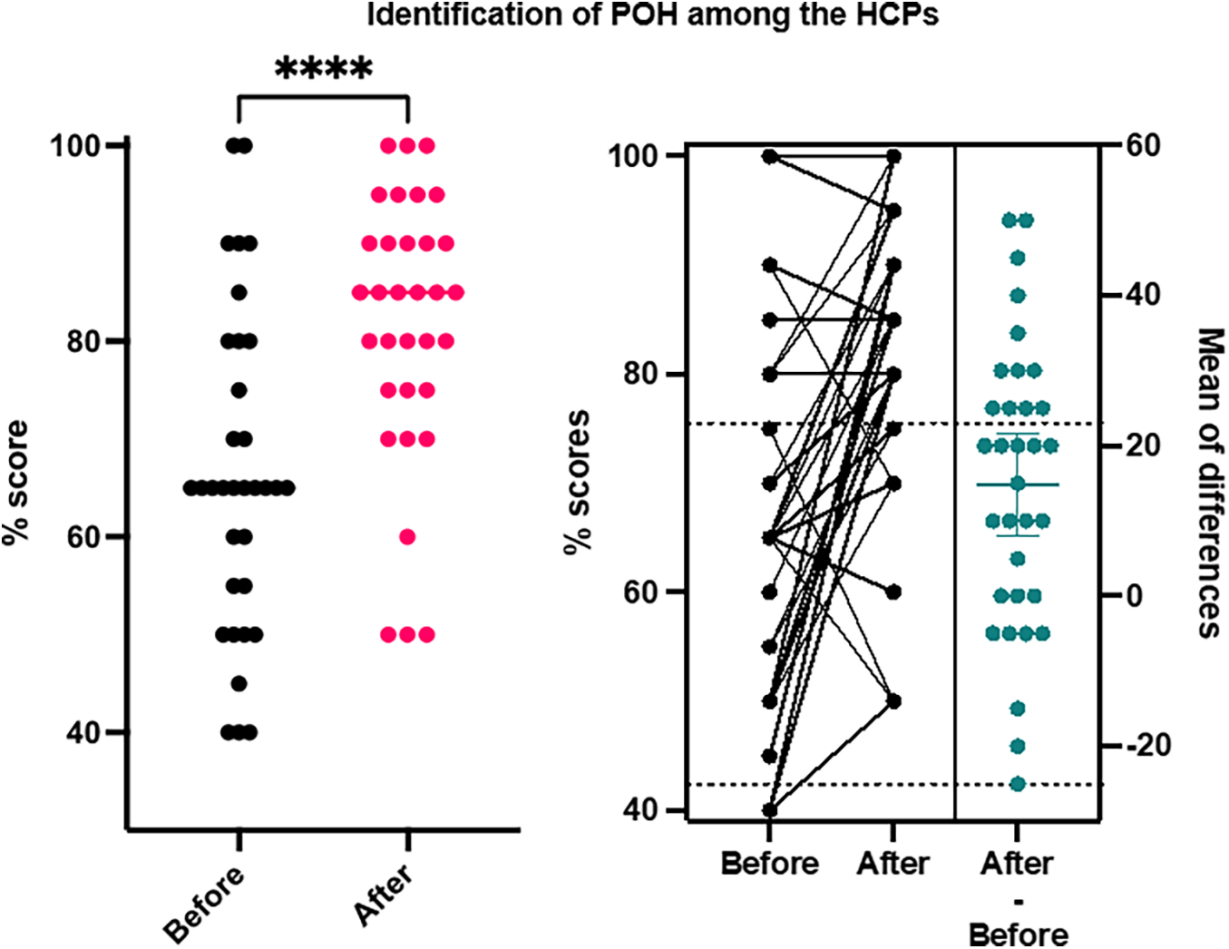
General proportion of the performance and distribution of the HCPs before and after training on identification of obstetric haemorrhage.

Of all participants, 32/33 (96.9%) passed the end-of-training final assessment (with 9 individuals repeating the assessment as they did not achieve the benchmark score of >75% on their first attempt) as shown in Figure 1. Of note, there were 6/33 (18.2%) who achieved a distinction score of >90% at the first sitting of the final assessment. These participants were invited to be program “champions” and identified as potential future local facilitators in the scale-up of the programme.

### Individual module assessment

**Figure 2:**
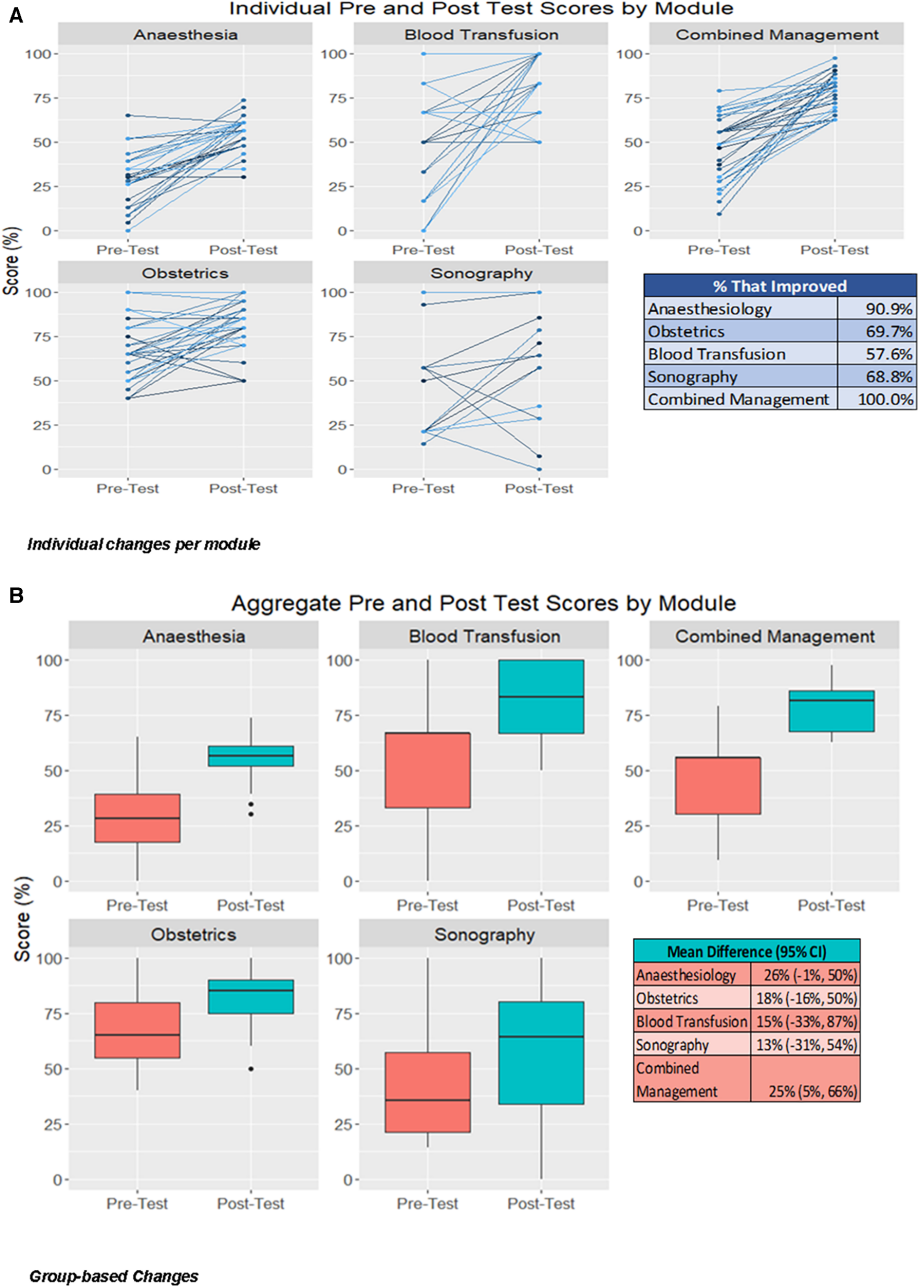
Individual and Group changes in all modules. (**A**) Individual changes (**B**) Group-based Changes.

Early sufficient antenatal identification of haemorrhage risk was recognised by 5 out of 34 (15%) participants in a written assessment before the training module and by 18 out of 33 (55%) participants after the module with maximum improvements among doctors (Figure 2) and Supplementary Figure S1.

In the anaesthesia module, the average score initially was 28.1% ± 15.6% ranging from 0% to 65.2% was improved to the average score of 55.3% ± 9.2% ranging from 30.4% to 73.9%. No one scored above 75% in the anaesthesia pre-training assessment before training and again no one scored above 75% in the post-training assessment.

With regards to obstetric surgical and medical interventions, there was an average score of 55.3 ± 9.2 ranging from 30.4 to 73.9 with 11 participants scoring > 75% in pre-module assessments. In the post-module assessment, 27 participants (81.8%) scored >75%. The average score initially was 66.3 ± 16.4 ranging from 40 to 100 which rose to 81.4 ± 13.8 ranging from 50 to 100, after training. score. The use of balloon tamponade increased from 11/34 (32.3%) to 26/33 (78.8), the use of the B-lynch suture from 11/34 (32.3%) to 25/33 (75.7), the need for internal iliac artery ligation and total abdominal hysterectomy (TAH) from 11/34 (32.3%) to 24/33 (72.7).

In the training for the choice of Whole Blood, Fresh frozen plasma and Platelets concentrate Transfusion initially, the average score was 54.9 ± 27.1 with only one out of 34 participants (2.9%) scoring at or above 75% before the module with the mean score 20.2, (95%CI of mean 17.53–22.9). After training the average score was 78.3 ± 18.8 ranging from 50 to 100 with 21 out of 33 HCPs presenting a score above 75% competency on Blood transfusion Supplementary Figure S2. The detailed distribution is shown in the Supplementary Figure S3.

About the ultrasound module, HCPs were surveyed before the training. 29/33 HCPs had access to or were already using ultrasound in their clinical practice. After the module, 15 participants reported that they would not access ultrasound in routine practice. In terms of knowledge improvement, three participants scored >75% in the pre-training assessment which improved to nine in the post-training assessment. Initially, the average score was 22.5% ± 30.5% and changed to 48.7% ± 33.9% (Supplementary Figure S4).

The combined knowledge presented an average score of 46.9% ± 17.8% ranging from 9.3% to 79.1% before training. The average score of combined knowledge after training was 78.4% ± 10.7% ranging from 62.8% to 97.7%. There were 21 out of 33 (63.6%) scoring above 75% cut point.

### Mean score assessment

There were 30/33 (90.9%) of HCPs improved Anaesthesia skills with a mean score improvement of 26% i.e., 0.26 (−0.009 −0.50). There were 23 HCPs (69.7%) who improved their skills on the use of the latest obstetric skills and Balloon tamponade and knowledge with a mean score improvement of 18% i.e., 0.18 (−0.16 to 0.50). There were 19 HCPs (57.6%) who improved competencies in Blood loss measurement and transfusion knowledge with a mean score improvement of 15%.i.e., 0.15 (−0.33 to 0.87). There were 20 out of 29 HCPs (68.8%) who improved Sonographical skills with a mean score improvement of 13%.i.e., 0.13 (−0.31 to 0.54). All 33 HCPs (100%) presented a combined change with the mean score improvement of difference of 25% i.e., 0.25 (0.05–0.66) as shown in Figure 2.

### Maternal mortality rates

Maternal mortality rates came down in hospitals at different levels of service provision with 95% significance (MZRH (*p* = 0.01), MRRH (*p* = 0.025), Mbalizi DDH (0.018) and Igawilo DH (*p* = 0.73. There were 32 deaths before training which reduced to nine deaths (*p* < 0.001) after the training. This represents a reduction in maternal mortality rates from 295/100,000 to 85/100,000 live births. For deaths attributed to obstetric haemorrhage, the mortality rate declined from 76/100,000 to 21/100,000 live births after implementation of the training (Table 2) which in absolute terms represents a decline from 8 to 3 maternal deaths due to obstetric haemorrhage in the month after training completed.

**Table 2:** Maternal mortality by hospital.

Maternal mortality by hospital											
	Pre-training (August 2021)					Post-training (April 2022)					
Hospital	Live births	Maternal deaths: cause PPH	Maternal deaths: cause other	Total maternal deaths	Total mortality rate (per 100,000 live births)	Live births	Maternal deaths: cause PPH	Maternal deaths: cause other	Total maternal deaths	Total mortality rate (per 100,000 live births)	*p*-value
MZRH	4,365	4	12	16	366.55	5,159	2	4	6	116.30	0.02
MRRH	1,671	2	4	6	359.07	3,127	1	3	4	127.92	0.1
Mbalizi DDH	1,990	2	5	7	351.76	2,431	0	1	1	41.14	0.03
Igawilo DH	2,479	0	2	2	80.68	3,455	0	1	1	28.94	0.58
Total	10,505	8	23	31	295.10	14,172	3	9	12	84.67	<0.001

Deaths due to other unspecified causes at the referral hospital, MZRH, dropped before and after training from 12 to 4, while deaths due to MOH halved from 4 to 2. At MRRH, deaths due to other unspecified causes dropped from 4 to 3 and deaths due to haemorrhage halved after training, albeit in small numbers (*n* = 2–*n* = 1). At Mbalizi DDH, no deaths due to haemorrhage were recorded after training (*n* = 2 before). At Igawilo District Hospital, no maternal deaths secondary to haemorrhage were recorded before or after training the detailed distribution is shown in the Supplementary Figure S5.

## Discussion

In this project, we were able to determine the effectiveness of a virtually-delivered, real-time, blended training programme designed for a range of HCPs who work with pregnant women in Mbeya, Tanzania. There was a training coverage of 30.8% of all HCPs in both Mbeya urban and rural public hospitals although there was a male majority which represented somewhat the doctor cadre. We were not powered to look at differences between female and male learners in this project The number of participants assessed meets the criteria for the 30 participants rule' for external validity of the Pre—Post studies (25). In this study, we were able to produce evidence of effective training methods to improve obstetric haemorrhage management in Mbeya, Tanzania.

The use of this well-coordinated blended training (London—Mbeya Project) provided evidence of competency among 32 of 33 HCPs who finished the training and passed the final assessment (96.9%). Knowledge and competency, as captured by standardised assessments in the 4 modules and the final simulation assessment were shown to have been significantly improved by completion of the course. In-service training has previously been shown to be effective in addressing obstetric haemorrhage at different levels of service provision in varied settings in Tanzania and sub-Saharan African countries (26).

Poor outcomes following obstetric haemorrhage are often attributed to lack of anticipation, delays in detection and prompt life-saving treatment (27). This study also reports a nearly 5-fold increase in the detection of bleeding risk. The project provided evidence of an increase in the identification of haemorrhage from 15% to 55% and an increase in combined obstetric haemorrhage management by 25%. This project feeds into strategies to ensure quality maternity care and Universal Health Coverage (UHC) (28, 29) that is demanded by the Tanzanian National Surgical, Obstetrics and Anaesthesia Plan (NSOAP) (30).

When assessing variances in baseline, improvement and end-line between different cadres of HCP recruited, it was apparent that our method provided a reduction in obstetric haemorrhage cases. We did not identify any major changes in personnel employed, equipment or drugs available throughout the study (reported through site visits and data collection) that would have directly influenced outcomes. The differences in knowledge seen between groups in the pre-module assessment disappeared after training. This means task shifting from surgeons, anaesthetists and obstetricians to non-physician clinicians (NPCs) and non-specialist physicians (NSPs) is possible when blended training is carried out as previously recommended by Falk and co-workers in 2020 (31). Our study highlights the opportunity to train the 4 modules in different cadres including non-physicians with a guided communication strategy in the fight against PPH. This finding needs to be tested in other settings in sub-Saharan Africa. From observations on simulation days, SBAR communication (24, 32) was a useful tool for communicating and handing over tasks between the cadres when Balloon Tamponade, B-Lynch sutures, choice and timing of blood transfusion were needed. In our training, SBAR communication provided sufficient guidance in task sharing needed for obstetric haemorrhage management (32).

The COVID-19 pandemic made it challenging to deliver this training in person by the UK team as originally planned. However, this presented an opportunity to develop a new virtual platform with real-time face-to-face local facilitation, which to our knowledge has not been used before in this setting. We found that an interactive web-based educational platform can provide a viable solution to training that could be extended to other medical fields and that can overcome barriers associated with traditional teaching methods, including cost and travel as shown in India (33). Additional benefits of the online modules format included the ability to recap content at one's own time and pace, improving the transferability of knowledge (34). Live delivery to groups facilitated discussion (both with the facilitator and each other) and enabled peer-to-peer learning. We found that it was possible to teach the practical skills, traditionally taught face to face, using a combination of our blended online approach and simulation, initially described by Sheen and colleagues (35) We have shown that blended training can have an impact (34, 36) in improving the management of obstetric haemorrhage.

We were also able to show a reduction in maternal mortality due to haemorrhage after the training was implemented in the 4 hospitals recruited. Although our study was not powered to detect a difference in mortality rates, this effect is worth exploring further through the up-scaling of this project. A recent randomised controlled trial (RCT) on a one-day course to reduce PPH found a reduction of severe PPH cases while case fatality did not improve (37). Our course was comprehensive, encompassing various aspects of PPH prevention, but more importantly, the training took place over several months which allowed for better consolidation and translation into a more sustained change in clinical practice and hence could explain this finding.

Given the small number of HCPs in this study, further evaluation and potential up-scaling of the blended training programme is required to validate our findings in broader contexts. The blended design makes it easily transferable and the use of local experts as co-leads can be recreated in other settings to help facilitate delivery of the programme. The model delivers “champion” HCPs identified during training, who can become future local trainers, enabling the up-scaling of training in the region and the maintenance of competencies. The next step of training would be a “train the trainers” course to ensure trainers are adequately equipped to deliver training so the initiative can become self-sustaining.

### Study limitations

The project was implemented during the COVID-19 pandemic which required the revision of some of the face-to-face simulation training planned. However, adapting to this meant we were able to deliver an almost entirely virtual course, although simulation training did require local face-to-face facilitators (with virtual training offered to these experts before the simulation day). The presence of local experts was necessary as the group had not previously undertaken simulation training and close guidance aided engagement with this component. With more experience and exposure to simulation training, local facilitation should be possible in any scale-up of the programme.

## Conclusion

Findings support further evaluation of the up-scaling of the training programme to other regions of Tanzania and other LMICs. The blended design of online and face-to-face training using local experts as co-leads to facilitate simulation-based training makes it easy to transfer knowledge and skills. The bringing together of multidisciplinary teams in local hospitals aided skills transfer and a sense of belonging. Evidence is provided that blended training can potentially help improve HCP knowledge and skills in the effective management of obstetric haemorrhage which has a critical role in reducing maternal deaths.

## Author’s note

The reflexivity statement for this paper is linked as the online Supplemental Files Figures 5. for the Obstetric project in Mbeya (London-Mbeya Project), Tanzania.

## Data Availability

The original contributions presented in the study are included in the article/**Supplementary Materials**, further inquiries can be directed to the corresponding author.
