# Supplementary figures and images for: The impact of a blended multidisciplinary training for the management of obstetric haemorrhage in Mbeya, Tanzania
Source: Front Glob Womens Health. 2023 Dec 7;4:1270261. doi: 10.3389/fgwh.2023.1270261 (PMC10748492; doi:10.3389/fgwh.2023.1270261)

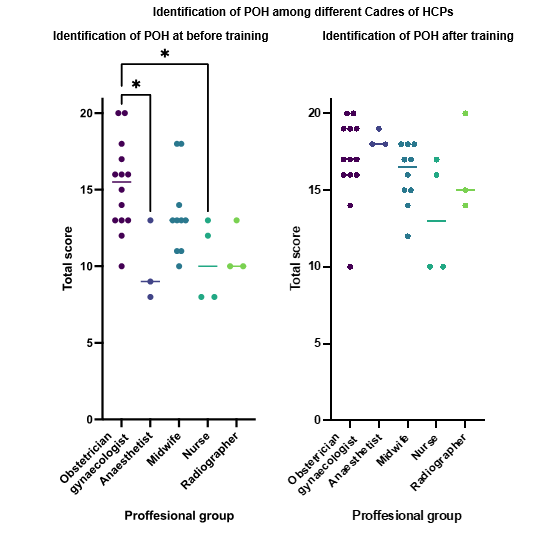

Supplement: Supplementary file 1 [file Image1.tif]

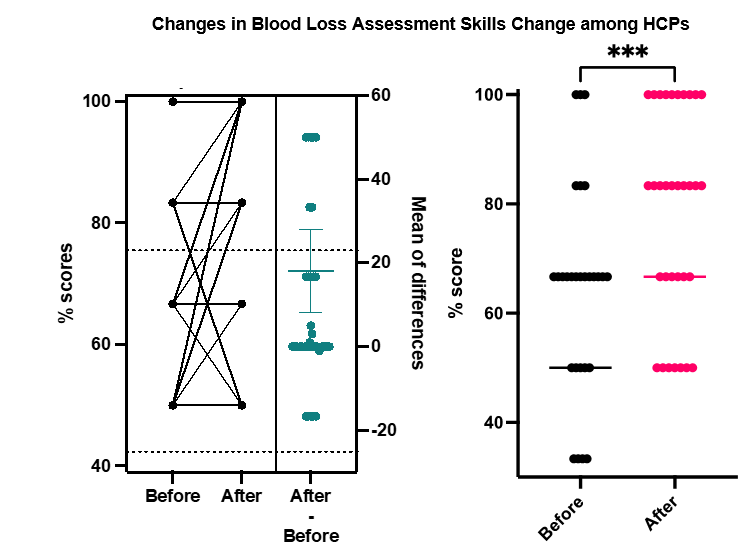

Supplement: Supplementary file 2 [file Image2.tif]

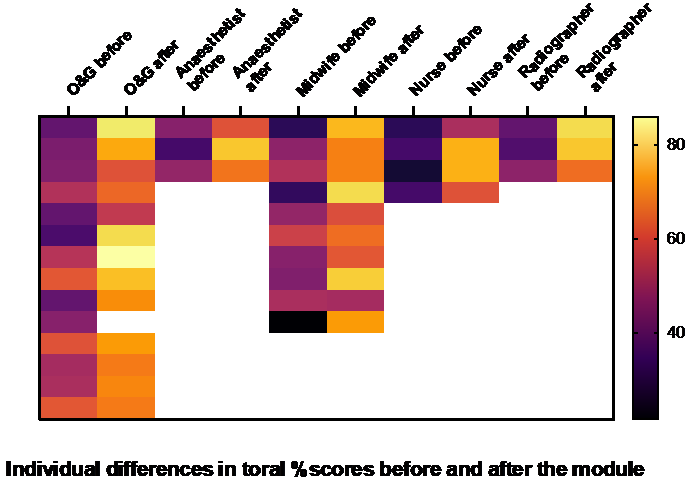

Supplement: Supplementary file 3 [file Image3.tif]

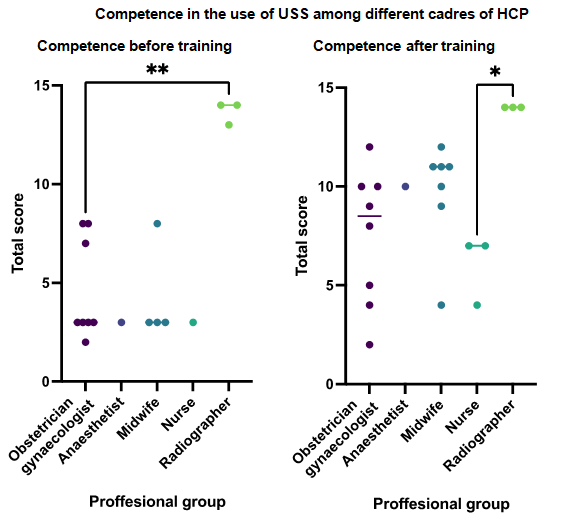

Supplement: Supplementary file 4 [file Image4.tif]

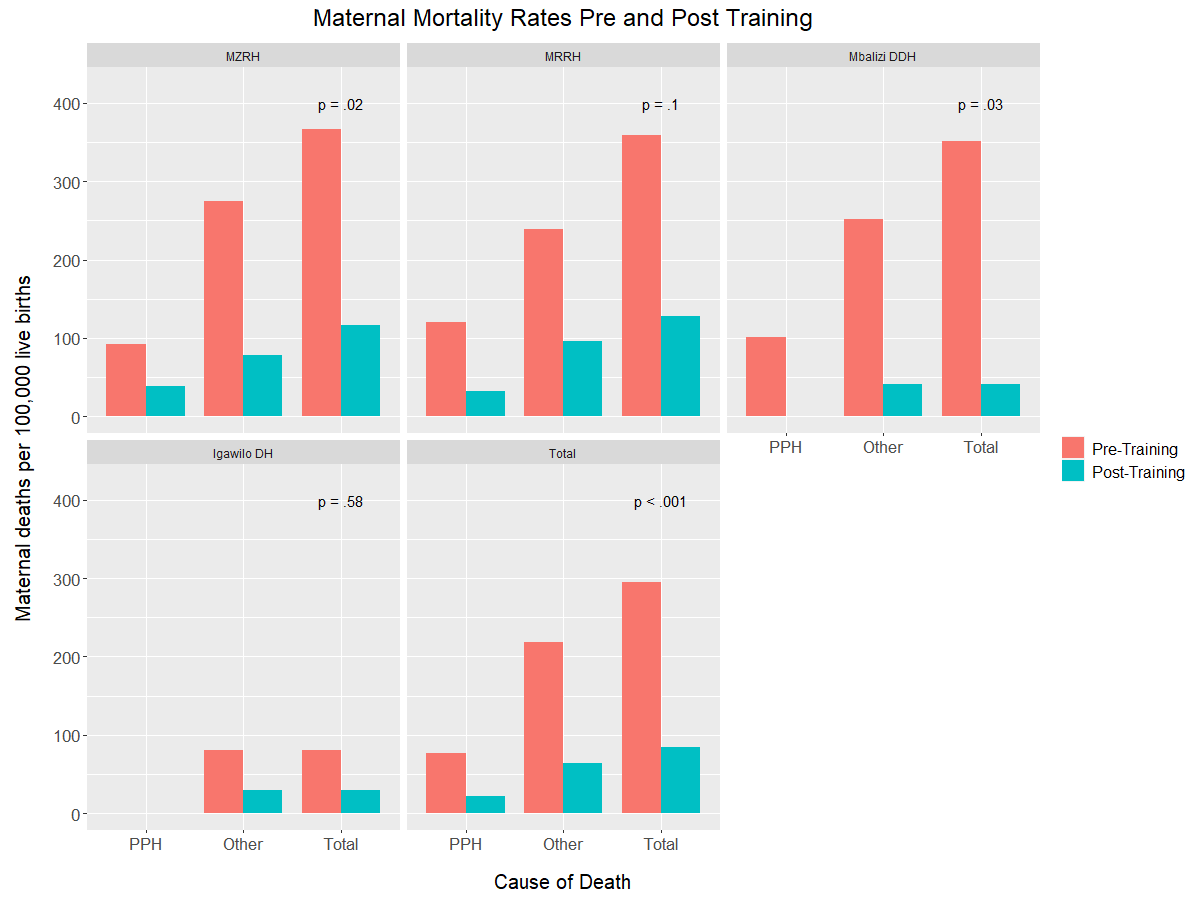

Supplement: Supplementary file 5 [file Image5.tif]
